# Foreign Summary

**Published:** 1840-04-11

**Authors:** 


### FOREIGN SUMMARY.

*Case of Diaphragmatic Hernia produced by a penetrating wound.* By John Reid, M. D., F.R.C.P.E., Lecturer on Physiology, President of the Anatomical Society, &c.—W. R. aged 45, a shoemaker, was admitted, on the 13th September, 1838, into the Infirmary, under the charge of Dr. Shortt, about noon. He stated that he had been seized about 4 o'clock, P. M. of the previous day with vomiting, succeeded by dyspnœa, and that the latter had continued to increase up to the time of his admission. He also stated, that for upwards of a year, he had been subject to occasional severe pain in the left hypochondriacal region, and also to cough without any expectoration. On admission he complained of great pain in the left hypochondrium, severe dyspnœa, with a depressed anxious expression of countenance; the pulse was intermittent, and so weak that it could not be reckoned. Extremities cold, and lips livid; percussion dull over the whole of the left side of the chest, and the respiratory murmur was there inaudible, while it was puerile on the right side. The sounds of the heart were only audible under the cartilages of the fourth, fifth, and sixth ribs on the right side. Had wine ordered, and sinapisms were applied to the chest and feet. Died in four hours after admission.*

*Sectio Cadaveris, 16th September.*—Heart healthy, but somewhat displaced towards the right side. Right lung sound. The left side of the chest contained more than six pounds of a reddish fluid, but there was no recent effused lymph on the pleura. The left lung was compressed towards the spine, and also towards the edge of the cordiform tendon of the diaphragm to which it adhered; and the left side of the diaphragm was pushed downwards into the abdomen. The lung itself was devoid of air. A dark soft mass was seen lying in the lower part of this side of chest, and was connected with the upper surface of the diaphragm. On examining the upper part of the abdomen, and the lower surface of the diaphragm, it was obvious that the dark mass observed in the chest was composed of a part of the transverse arch of the colon, and a considerable mass of the large omentum, which had passed through an opening with callous edges, in the diaphragm, and become strangulated. The parts were now carefully examined *in situ*. A cicatrix nearly half an inch in length was observed in the skin at the lower part of left side of chest, midway between the anterior and poste-

* The above account of his symptoms on admission was furnished by Dr. Alexander Wood.

rior extremities of the ribs; and, on dissecting off the skin, a similar cicatrix was found in the ninth and tenth intercostal space, exactly opposite to that in the skin. The same cicatrix was distinctly seen on looking at the inner surface of the chest, and a process of that part of the large omentum which had accompanied the transverse arch of the colon through the diaphragm, not only adhered to the edge of the cicatrix, but was incorporated with it, and projected into the intercostal space. The aperture in the diaphragm would have admitted the passage of the points of three fingers with difficulty, and was filled up by the protruding and returning portions of the transverse arch of the colon, and was situated between the last left rib and cordiform tendon of the diaphragm, and between two and three inches from the origin of the muscular fibres of the diaphragm from the last rib, and was on a line with the cicatrix in the intercostal space, but placed on a lower level when the diaphragm was depressed. On the other hand, when this muscle was pushed up, in a manner similar to what must occur, when it is in a relaxed state, or in the act of expiration, the opening in the diaphragm was then brought on a level with the cicatrix in the intercostal space, and considerably approximated to it. That part of the transverse arch of the colon which lay within the chest was at least a foot in length, was considerably dilated, and much thickened in its coats, was of a deep dark colour, was soft in several places, and at one point had given way. The two portions of the transverse arch of the colon which lay in the opening of the diaphragm were considerably constricted. The entering portion (or the portion next the *caput cœcum*) could still be drawn upwards and downwards, and the little-finger introduced into the interior of the tube could be pressed upwards, though with some little difficulty, through the opening in the diaphragm. The returning portion of the transverse arch of the colon was connected to the margin of the opening in the diaphragm through old adhesions of the omentum.*

It was stated by a friend of this man, who was present at the inspection, that he had received a wound about fifteen months ago, in the lower part of left side of chest with a shoemaker's knife, in a quarrel with a woman with whom he cohabited, and that she was liberated from prison after his apparent recovery. Between the time of his receiving the wound and his death, it appears that he was at two different times a patient in the Infirmary with symptoms of severe ileus, and was at each time dismissed apparently cured.

Though an examination of all the circumstances connected with this case leads us to the conclusion, that the protrusion of the transverse arch of the colon into the cavity of the left pleura took place in consequence of a wound made in the diaphragm, and that this was the cause of death, yet there are various considerations, arising from the accounts given by authors of cases where a greater or less number of the abdominal viscera had passed into the thorax from a congenital deficiency in the diaphragm, which ought to be carefully weighed before we can venture to give a definite opinion in similar occurrences, as this may involve most important consequences. Doubts will, and ought, under such circumstances, to exercise an amount of influence on our decisions, which we might not be disposed to allow them in ordinary cases. The fact, that the omentum not only adhered to the edges of the cicatrix of the wound observed on the inner surface of the thorax, but was also incorporated with it, and projected into the intercostal space, is sufficient to prove, that the omentum was present in the left side of the chest during the time that the wound was healing; but it is not of itself sufficient to decide the question which may be raised, as to the possibility of the passage of the intestine into the chest at some period previous to the infliction of the wound.

1. The first consideration which naturally presents itself on examining the recorded cases of the passage of some of the abdominal viscera into the thorax from congenital deficiency of part of the diaphragm, is the circumstance, that, though a considerable number of these have been observed in infants who were either still-born, or who died within a few weeks after birth, and had oppressed breathing from the moment of birth up to their death; yet there are others where the individual lived several years, and in some cases even to an advanced age, without any suspicion having been entertained that such a displacement of the abdominal viscera existed. In some of these, however, as we shall immediately see, the respiratory function was more or less embarrassed.

Riverius* relates the case of a young man, who, when slowly convalescing from intermittent fever, took an antimonial emetic from an empiric, and after ineffectual efforts to vomit, died some hours after. On dissection, the stomach was found in the right side of the chest, and the lung of that side was defective. Up to the time of his death, this young man had experienced no dyspnœa, had enjoyed good health up to the period of his last illness, and had served as a soldier. G. T. Weyland† has minutely detailed the case of a boy seven years of age, who had been affected with frequent vomiting from his infancy. He appeared to have no other complaint; but this, from

* The preparation is preserved in the University Museum.

* Opera Omnia Medica. Observ. Centuria Quarta, Observ. 67, p. 549. Lugd. 1690.

† Dissertatio Inauguralis Medica Duos Exhibens Casus Dislocationis Viscerum nonullarum Abdominis. Jenae, 1831.

its frequency, rendered his body thin and imperfectly nourished. He was not observed to have any difficulty of breathing, even when playing with his companions. When he was 7 years of age, the vomiting became so frequent, accompanied by pain of head and abdomen, and his health was suffering so much, that a physician was sent for. After being subjected to treatment for about twelve days, he began slowly to recover his strength. Fourteen days after this, he had a relapse after exposure to cold, and he died. On removing the sternum, the left side of the chest was seen to be filled with the folds of the intestines as high as the second rib, aud the left lung was consequently much diminished in size, and devoid of air. The stomach was placed in the abdomen, was of great size, and lay in a vertical position, its pyloric extremity extending downwards to the pelvis. Dr. Monro, *Tertius*, has detailed two very interesting cases of this kind. One was a female 22 years of age, who died with symptoms of internal strangulation; and on inspection of the body, a large portion of the arch of the colon was found to have passed through a small opening in the left side of the diaphragm. She never had any difficult breathing, but was subject to pain in the lower part of the left side. The other was a male of middle age, who died with cerebral symptoms, accompanied with difficult breathing and expectoration, and the arch of the colon and the omentum were found in the left side of the chest. He had an attack of ileus four months previous to his death.* Bartholin† and Clauder‡ relate the case of a man who had always enjoyed a free and easy respiration, except a temporary fit of asthma, but who had long laboured under vomiting and constipation, in whom the stomach, the duodenum, part of the colon and omentum, had passed through a large opening with callous edges into the left side of the thorax. Petit§ details the case of a man who had cholic pains and difficult breathing for forty years, and on dissection, a great portion of the colon, of the omentum, and of the splenic extremity of the stomach, had passed into the left side of chest. The protruded parts had contracted no adhesions, and were not covered by a hernial sac. Sir Astley Cooper|| relates the case of woman, who died, when 28 years of age, with symptoms of inflammation of the abdomen of a few days standing. On dissection, about 11 inches of the transverse arch of the colon, and a great part of the omentum, had passed through an opening in the diaphragm into the left side of the chest, and the omentum adhered to the aperture in the diaphragm. Dr. John Clark* details the case of a man who died, when 40 years of age, with all the symptoms of peritoneal inflammation, and on dissection, a part of the transverse arch of the colon, part of the left lobe of the liver passed through an opening in the diaphragm, which he supposed to arise from congenital malformation of that muscle. This man had enjoyed good health up to the two last years of his life. As he had received a fracture of two of his ribs a year before his death, it may be argued that a rupture of the diaphragm may have occurred at that time; yet, as it is stated that he recovered from the injury without any unusual symptoms, the case may at least be adduced as additional evidence of the fact, that part of the abdominal viscera may be present in the chest without any symptom to indicate their presence. Chauvet† states, that on examining the body of a lieutenant-colonel, he found the stomach and colon in the left side of the chest. And Vetter‡ observed in a very old person the whole tract of the small intestines lying in the left side of the chest.§ Along with the above cases we may also include other three, in which part of the abdominal viscera appear to have protruded upwards into the thorax through the interval which naturally exists between the outer edges of the muscular fibres of the diaphragm which arise from the ensiform cartilage of the sternum, and those which arise from the cartilages of the ribs. One of these is related by Morgagni.|| It occurred in a man about 50 years of age, who died from a fracture of the skull, and the protruding part was the colon. A second case is related by Sir Astley Cooper,¶ on the authority of Mr. Bowles, and in this the right extremity of the stomach, the beginning of the duodenum, and part of the omentum, protruded into the right side of the chest, and were covered by a hernial sac formed by the peritoneum and the pleura. This man was also fifty years of age, and subject to asthmatic attacks, and died from excessive vomiting, after the exhibition of an emetic, as occurred in the case related by Riverius,**

* Morbid Anatomy of the Gullet, Stomach, and Intestines, p. 180. 2d edition.

† Boneti Sepulch. Anat. Tom. ii. p. 803.

‡ Vide Sepulchretum, et Morgagni de Sedibus et Causis Norborum, Lib. iv. Epist. 54. § 11.

§ Malades Chirurgicales, Tom. ii. p. 161. 1783.

|| Medical Records and Researches. London, 1796.

* Transactions of a Society for the Improvement of Medical aud Chirurgical Knowledge, Vol. ii. p. 118.

† Histoire de l'Academie Royale des Sciences de Paris for 1729, p. 14.

‡ Aphorismen aus der Pathologischen Anatomie, S. 144, as quoted by Weyland.

§ Chauvet says nothing about the state of the respiratory and digestive organs during life in the case which he relates; and Weyland does not inform us whether the previous history of the case by Vetter, was ascertained.

|| De Sedibus et Causis Morborum, Lib. iv. Epistol. 54, § 11.

¶ Opus cit. p. 14.

** Petit (opus cit.) relates the case of a man

Another case, where the diaphragmatic hernia protruded through the interval on the right margin of the fibres arising from the ensiform cartilage, is related by Professor Bignardi.*

After a knowledge of the above cases, it could not be argued that, if the protrusion of the transverse arch of the colon, in the case we have detailed, had arisen from congenital deficiency in the diaphragm, it must have manifested itself before the reception of the wound, by some embarrassment of the respiratory function. The fact, moreover, that this protrusion must have existed from the time of the infliction of the wound up to his death, without any marked impediment to the respiration, is obviously quite sufficient to invalidate any similar argument.

2. In the case we have related the protrusion of the colon through the diaphragm had occurred on the side of the chest, in which it is generally found in cases of deficient formation of the diaphragm. Of twenty-four recorded cases (not including the three last referred to,) which I have examined for this purpose, I find that in three only had the protrusion happened on the right side. One of these is the case of Riverius, already referred to; another is one of the two cases mentioned by Dr. Macauly,† and one by Bonn.‡

3. The congenital deficiencies in the left side of the diaphragm, not only vary in extent, but also in position. In several of the recorded cases, the deficiency appears to have included the whole of the left side of the diaphragm, and in others it is merely stated that the opening was on the left side. In two cases the opening was placed in the cordiform tendon, (case of Petit, and one of the cases of Weyland;) in one it was stated to have been near the opening for the *cava ascendens*, (one of Vetter's cases;) in one the protrusion is said to have occurred through the opening for the sympathetic nerve, (Platner's Disput. De Hydrocel, as quoted by Morgagni;) in three the protrusion occurred through the œsophageal opening (Resigius de Ventriculi in Cavo Choracis Situ Congenito, Berlin, 1823;) a case by Fantoni, as quoted by Monro, and a case by Clauder; in one it was placed an inch to the left of the œsophageal opening, (one of Dr. Macauly's cases;) in one, it was placed three inches to the left of the œsophagus, (case of Sir A. Cooper;) in one, in the middle of the left portion of the diaphragm, (one of Monro's cases;) and in one (supposing it to be a case of congenital hernia) it must have occupied nearly the same situation as in the case we have described, since it is stated to have been situated "about three inches from the ribs, and placed more anteriorly than the œsophagus."

4. In at least two of the recorded cases, the protruding parts were firmly adherent to the edges of the opening, (cases of Sir A. Cooper and Chauvet.)* We would certainly expect that in cases of wound of the diaphragm, that if the person survived, inflammation of the edges of the divided muscle would occur, and probably cause adhesion between the edges of the wound and the parts which may have protruded into it, as in the case we have described. That lymph may be effused and unite the protruded parts to the edges of the opening in some cases of congenital deficiency of a part of the diaphragm, is, *a priori*, certainly quite possible, and in two cases which we have cited above it had actually occurred.

5. Some of the cases of congenital deficiency of the diaphragm terminated fatally, as in ours, by strangulation of the protruding portion of the intestines, (cases of Dr. Clark, Sir A. Cooper, and Monro.)

Approaching the consideration of this case with all the caution which an examination of the recorded cases of congenital malformation of the diaphragm must naturally induce, we still feel strongly convinced that the protrusion of the colon was the consequence of a wound of the diaphragm. If we reflect upon the circumstance, that a sharp instrument could scarcely be thrust into the chest through the ninth and tenth intercostal space, where the cicatrix was found, without wounding the diaphragm, and thus necessarily at the part where the opening in it was found, if the muscle be in a relaxed state, or during expiration, as we have frequently satisfied ourselves upon the dead body; upon the great rarity of such small congenital deficiencies in the diaphragm; upon the uneasiness felt by the patient in the left hypochondrium, and the severe attacks of ileus after the infliction of the wound; conjoined with the improbability that a person could have arrived at the age of 45 without fatal strangulation of the intestine, and have acquired an athletic form of body, when that part of the intestine passing through the opening would with difficulty admit the passage of the little finger, even when the returning portion was empty,—we can scarcely suppose that the conjunction of the opening in the diaphragm and the infliction of the wound in the parietes of the chest, were in this individual a mere colong subject to dyspnœa, and who had been treated for asthma, in whom the stomach, the colon, and the omentum were pushed up into the left side of the chest, and were enclosed in a sac formed by the diaphragm, peritoneum, and the pleura. See also Beclard's Supplement au Traite de Scarpa, p. 132, for two cases where the sac was formed by the pleura and peritoneum.

* Sull' ernia diaphragmatica, 1827, as quoted by Laurence.

† Medical Observations and Inquiries, Vol. i. p. 26, London, 1763.

‡ Descriptio Thesauri Ossium Morbosorum Hoviani, No. 204, p. 69, as quoted by Laurence.

* In Sir A. Cooper's case, the omentum was the adherent part.

incidence. It ought not, however, to be concealed, that a more accurate account of the previous history of the patient than we are able to give would have been very desirable.

Believing that the protrusion of the intestine into the chest was in this case the consequence of a wound in the diaphragm, there are some cases on record which bear to it a greater or less resemblance; and it is of some importance to know, that the accounts given of them do not discountenance the idea we have formed of the nature of this case. Sennertus relates, in a letter to Hildanus,* the case of a man who stabbed himself beneath the ninth left rib, and died about seven months afterwards. On dissection the stomach was found to have protruded through the cordiform tendon into the left side of the chest.† In this case the seat of the wound in the parietes of the chest must have been placed nearer the sternum than in the one we have detailed. Ambrose Paré‡ relates the case of a person who died eight months after receiving a penetrating wound in the chest, and in whom a large portion of the colon was found in the cavity of the left pleura. This person had suffered from colic pains after the reception of the wound. Mr. Boyle § gives the case of a soldier who received a wound in the chest eleven months before his death. He died with symptoms of peritoneal inflammation, and a great part of the ileum and transverse arch of the colon had passed through an opening in the diaphragm, and became strangulated. From the time he received the wound "the respiration was affected, and even moderate exercise was supported with difficulty." Mr. Greetham‖ has published a case of a muscular man, between 30 and 40 years of age, who had been wounded by a knife some years before in the left side of the chest, and who died with all the symptoms of strangulated intestine. On dissection, the omentum and a part of the colon had protruded through an opening in the cordiform tendon of the diaphragm. He had at times severe pains in the stomach after the infliction of the wound; but he had been able to act as a steward in a vessel during several distant voyages. The larger curvature of the stomach was drawn upwards towards the opening in the diaphragm by the omentum in the chest, so that it was nearly reversed.

Dr. M'Crie¶ has given an account of a preparation preserved in the Chatham Museum, obtained from a soldier who had been wounded twenty-two years before, and who died of gangrene of the lower extremities. In this case the stomach and a great part of the transverse arch of the colon had passed into the chest. After the reception of the wonnd up to the time of his death, he laboured under dyspeptic symptoms, and his breathing was affected on walking fast, or on ascending a hill, but these did not prevent him from acting as a sergeant in his regiment.

It would also appear that similar effects may result from laceration of the diaphragm from a fall. Dessault* relates the case of a man who received a fall when 39 years of age, and who lived four years after this, in whom the stomach and arch of the colon had passed through an opening with callous edges into the left side of the chest. Though he was able to return to his usual employment after his recovery, he continued to complain of pain of chest and oppression of the breathing. Mr. W. D. Morgan has described the history of a patient who died in the Bristol Hospital, in whom several parts of the alimentary canal were found within the left side of the chest. The opening in the diaphragm was attributed to a fall upon his back received thirty-eight years before. From the time of the injury to his death, he was able to follow his usual occupation as a mason, but had often suffered from asthmatic dyspnœa, dyspepsia and constipation.† A very remarkable case is related by Mr. Taylor,‡ of a man who had received many years before his death, a fall in which some of the left ribs were fractured. On dissection, a great part of the left side of the chest was filled by the stomach, and a large part of the transverse arch of the colon, yet he was able to perform the usual duties of a sailor up to his last illness, and the respiration appears to have been little affected. This man died after amputation of a leg.

*Ed. Med. and Surg. Journ. Jan.*, 1840.

---

*The Army.*—Of the value of the two classes as soldiers, recruits from the agricultural class are held in the Army to be more trustworthy than those from trades and manufactures. Agricultural labourers generally enter the Army in consequence of some family difficulty or discord; while recruits who are enlisted in the manufacturing districts are frequently idle and dissolute, and require all the means in the power of officers to correct their intemperate and vicious habits. Mr. Marshall mentions, as an evidence of the improved condition of the people, that in one district, upon an average, from one-fourth to one-third who enlist, pay the smart-money, and all the other expenses which have been incurred on their account, rather than complete the enlistment before a magistrate.

*London Lancet.*

---

* G. F. Hildani, Medico-Chirurgi, Obser. et Curat. Chirurg. Centuriae. Cent. ii. Observ. xxxiii. 1541.

† Vide Morgagni de Sedibus et Causis Morborum, Lib. iv. Epistol. 54, §. 12 for other cases.

‡ Lib. 9, Cap. 30.

§ Vol. viii. of this Journal.

‖ Medical Gazette, Vol. x. p. 43.

¶ Medical Gazette, Vol. xv. p. 872.

* Journal de Chirurgie per Dessault, Tom. iii.

† Medical Gazette, Vol. xii. p. 673.

‡ Guy's Hospital Reports, Vol. iii. p. 366.

